# Dopamine in the Nucleus Accumbens Signals Salience of Auditory Deviance

**DOI:** 10.1111/ejn.70486

**Published:** 2026-04-02

**Authors:** Riko Iizuka, Ryotaro Yamaki, Tomoyo Isoguchi Shiramatsu, Shota Morikawa, Yuji Ikegaya, Hirokazu Takahashi

**Affiliations:** ^1^ Graduate School of Information Science and Technology The University of Tokyo Tokyo Japan; ^2^ Graduate School of Science The University of Tokyo Tokyo Japan; ^3^ Graduate School of Pharmaceutical Sciences The University of Tokyo Tokyo Japan; ^4^ Institute for AI and Beyond The University of Tokyo Tokyo Japan; ^5^ Center for Information and Neural Networks, National Institute of Information and Communications Technology Osaka Japan; ^6^ International Research Center for Neurointelligence (WPI‐IRCN), Institutes for Advanced Study (UTIAS) The University of Tokyo Tokyo Japan

**Keywords:** deviance detection, dopamine, nucleus accumbens, oddball, salience

## Abstract

How the brain signals prediction errors for non‐rewarding, yet significant, sensory events remains a central question. Although the cortical mismatch negativity provides a well‐known signature for deviance detection, the contribution of subcortical dopamine remains unclear. This study tested the hypothesis that phasic dopamine in the nucleus accumbens encodes the salience associated with the violation of an ongoing statistical regularity. Using fiber photometry in freely moving rats, we contrasted an auditory oddball paradigm with a many‐standards control. Deviant stimuli elicited a significantly amplified dopamine response compared with standard stimuli. Crucially, this dopamine response enhancement was absent in the control condition, demonstrating that the nucleus accumbens dopamine responds specifically to rule violation rather than mere stimulus rarity. The long latency of this signal (~500 ms) relative to the cortical mismatch negativity argues against a direct role in the initial detection of deviance. Instead, our findings support a model in which subcortical dopamine acts as a distinct salience signal, operating in parallel with cortical deviance detection, to evaluate unexpected events and guide subsequent behavioral adjustments.

## Graphical abstract

Using fiber photometry in freely moving rats, we show that nucleus accumbens dopamine signals the salience of violations of ongoing auditory regularities. Deviant sounds in an oddball sequence evoked larger dopamine responses than physically identical rare sounds in a many‐standards control, dissociating rule violation from rarity. The ~500 ms latency suggests a downstream precision‐like signal complementing cortical MMN.

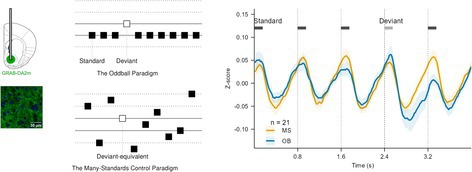

**Abbreviations**
AAV: adeno‐associated Virus
AP: anteroposterior (stereotaxic coordinate)
BF: Bayes factor
DA: dopamine
dB SPL: decibels sound pressure level
Dev: deviant
DV: dorsoventral (stereotaxic coordinate)
Fig.: figure
GRAB: GPCR‐activation‐based (sensor)
hSyn: human synapsin (promoter)
ML: mediolateral (stereotaxic coordinate)
MMN: mismatch negativity
MS: many‐standards (control paradigm)
NA: numerical aperture
NAc: nucleus accumbens
OB: oddball (paradigm)
PBS: phosphate‐buffered saline
PET: positron emission tomography
PFA: paraformaldehyde
PI: Preference Index
SEM: standard error of the mean
SOA: stimulus onset asynchrony
Std: standard

## 1 Introduction

Detecting unexpected sensory stimuli is crucial for adaptive behavior. This process, known as deviance detection, is often studied using the oddball paradigm, where rare deviant stimuli interrupt a sequence of repeating standard stimuli (Näätänen et al. 1978). The oddball paradigm reliably elicits the mismatch negativity (MMN), a cortical response to the violation of an ongoing statistical regularity. MMN is considered a neural marker of sensory prediction error within the predictive coding framework (Bastos et al. 2012; Carbajal and Malmierca 2018; Friston 2005; Garrido et al. 2009). Prediction errors detected in the cortex are subsequently processed along hierarchical pathways (Bastos et al. 2012; Parras et al. 2017).

For a prediction error to lead to an adaptive behavior, its behavioral salience must be evaluated. Salience comprises multiple dimensions: physical factors (e.g., intensity), motivational value (reward/punishment), contextual relevance, and attributes such as novelty (Bromberg‐Martin et al. 2010; Schultz 2013). Indeed, the magnitude of the MMN is modulated by prior experience, including learning and context, providing direct evidence that deviant stimuli in an oddball paradigm possess empirical salience (Chang et al. 2017; Fitzgerald and Todd 2020; Knudstrup et al. 2025; Shiramatsu and Takahashi 2018). The mesolimbic dopamine system is thought to evaluate these diverse forms of salience (Bromberg‐Martin et al. 2010; Horvitz 2000; Schultz 2016). Phasic dopamine signals encode not only reward prediction error but also novelty and physical stimulus properties (Comoli et al. 2003; Dommett et al. 2005; Gonzalez et al. 2023; Kutlu et al. 2021; Legault and Wise 2001; Sadacca et al. 2016). This evidence has given rise to the theoretical framework that dopamine contributes to adjusting the ‘precision’ of prediction errors, thereby regulating learning. Within the predictive coding account, dopamine is proposed to signal the precision (i.e., reliability or certainty) of prediction errors, modulating synaptic gain and learning rates (Friston 2005; Friston et al. 2012, 2015; Stephan et al. 2006). From a motivational perspective, dopamine is further viewed as dynamically evaluating whether an event is worth allocating limited resources such as attention and energy (Berke 2018; Hamid et al. 2016; Mohebi et al. 2019; Salamone et al. 2007).

Clinically, aberrant salience in schizophrenia is associated with dopaminergic dysfunction and attenuated MMN, suggesting a functional link between the sensory cortical prediction‐error processing and dopaminergic signaling (Avissar et al. 2018; Baldeweg et al. 2004; Erickson et al. 2016; Umbricht and Krljes 2005). Yet, pharmacological evidence remains inconsistent; MMN exhibited partial dependence on dopamine levels in rodents (Inaba et al. 2021; Valdés‐Baizabal et al. 2020), but not in humans (Kähkönen et al. 2002; Korostenskaja et al. 2008; Leung et al. 2007, 2010). This discrepancy, particularly the null findings in human studies, suggests that dopamine is not required for the initial generation of cortical MMN. Instead, it raises the possibility of a parallel processing model, in which deviance elicits two complementary processes: a rapid cortical computation of prediction error and a subcortical evaluation of salience. Although the cortical MMN is well characterized, the nature of the corresponding subcortical dopamine signal remains poorly defined. To our knowledge, no prior study has directly characterized dopaminergic responses in a classic MMN‐evoking paradigm using an oddball versus many‐standards control contrast.

This gap in the literature likely reflects a combination of historical and methodological factors. First, there has been a temporal‐resolution mismatch; the MMN is a fast cortical response occurring on a sub‐second scale (Fitzgerald and Todd 2020; Inaba et al. 2021; Näätänen et al. 2007), whereas traditional dopamine measurement techniques (e.g., microdialysis, PET) operate on a timescale of seconds to minutes. This technological limitation has only recently been overcome with the advent of genetically encoded dopamine sensors (e.g., GRAB‐DA) and fiber photometry (Beyene et al. 2018; Sabatini and Tian 2020; Simpson et al. 2024; Sun et al. 2020). Second, paradigm‐related challenges exist, as the subtle acoustic deviations used in classical oddball paradigms often carry weak behavioral relevance, making it difficult to elicit a clear dopaminergic response. Third, a persistent division between research communities—cortical electrophysiology in MMN research versus reward and motivation in dopamine research—has hindered the integration of theoretical frameworks and experimental techniques.

This study advances and empirically tests the unifying hypothesis that a deviant event triggers parallel processing within cortical and mesolimbic systems. We hypothesize that although the cortex computes a prediction error signal (the MMN), the mesolimbic dopamine system performs a parallel evaluation of salience—a signal that corresponds to the theoretical concept of precision in the predictive coding framework (Friston et al. 2012, 2015; Haarsma et al. 2021). An alternative possibility is that dopamine first detects deviance and then broadcasts a salience signal upstream to the sensory cortex. This non‐parallel account predicts earlier dopaminergic transients than cortical mismatch responses and direct dopaminergic modulation of cortical deviance signals, beyond effects of rarity or physical salience. To adjudicate between these accounts, we combine a classical oddball paradigm with a many‐standards control, which dissociates rule violation from stimulus rarity and sensory adaptation. This design directly tests whether the violation of an ongoing statistical regularity modulates the dopamine response. In addition, we systematically manipulate stimulus intensity and duration to functionally dissect the component processes of the dopaminergic response.

The central prediction of this study is that the nucleus accumbens (NAc) dopamine responses to deviant stimuli in the oddball paradigm will be significantly greater than those to standard stimuli, as well as greater than those to physically identical stimuli in the many‐standards control paradigm, and that this dopaminergic signal will be delayed relative to the cortical MMN. If confirmed, this pattern would provide evidence that dopamine does not contribute to the initial generation of the cortical deviance signal but instead functions as a distinct salience signal, operating in parallel with cortical detection of deviance to evaluate the behavioral significance of unexpected events. By explicitly bridging the two major research fields of predictive coding and reinforcement learning, this study aims to elucidate a more general neural principle for how the brain evaluates the salience of surprising events.

## 2 Materials and Methods

### 2.1 Animals

This research was conducted in full compliance with the “Guiding Principles for the Care and Use of Animals in the Field of Physiological Science,” established by the Japanese Physiological Society. The Committee on the Ethics of Animal Experiments and on the Biosafety at the Graduate School of Information Science and Technology at the University of Tokyo authorized the experimental protocols (A2024IST002, G2024IST002). All surgical procedures were performed under isoflurane anesthesia, and every effort was made to minimize pain and distress. After the experiments, animals were euthanized by an intraperitoneal overdose of pentobarbital sodium (160 mg/kg).

### 2.2 Sound‐Preference Assay

**FIGURE 1:**
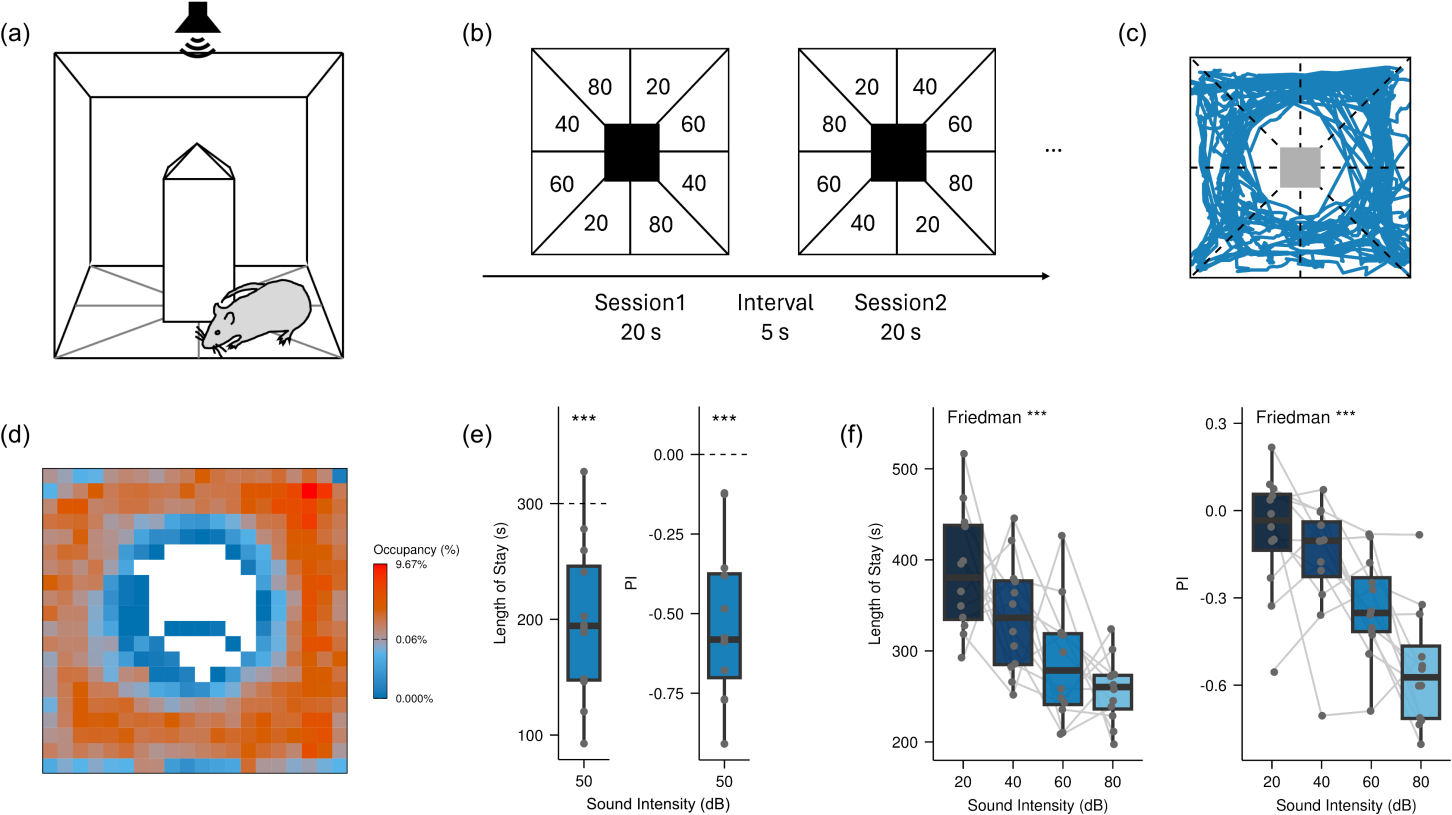
Behavioral assessment of aversion to white noise. (a) Experimental setup. Auditory stimuli were delivered contingent on the rat's position within an arena virtually divided into eight fields surrounding a central pillar. (b) Representative field‐intensity mappings for the 20–80 dB SPL experiment. Sessions were repeated with a 5‐s inter‐session interval, during which the field‐intensity mapping was shuffled (25 sessions for 0/50 dB SPL experiment or 50 sessions for 20–80 dB SPL experiment). (c) Example trajectory of a single rat aggregated over multiple sessions. (d) Cumulative heatmap for all the animals in 20–80 dB SPL experiment. Occupancy was binned into a 20 × 20 grid, with the color scale indicating the percentage of total recording duration. The gradient is displayed on a pseudo‐logarithmic scale (base = 10, *σ* = 0.001). (e) Preference metrics for the 50‐dB SPL versus 0‐dB SPL white noise experiment (*n* = 12, 25 sessions). Length of stay (left) and Preference Index (PI; right) are shown. The dashed line indicates the median stay duration (300 s), and 0.00 PI represents chance level (no preference). Rats spent significantly less time in the 50‐dB SPL field (*p* < 0.001) and showed a significant negative PI (*p* < 0.001). (f) Intensity‐dependent aversion in the 20–80 dB SPL experiment. Length of stay (left) and PI (right) are plotted as a function of sound intensity (*n* = 12, 50 sessions). Aversion, indicated by shorter stays and lower PI, increased significantly with sound intensity (*p* < 0.001 for both metrics).

Rats' preference for white noise was assessed according to Soga et al. (2018) (*n* = 12). Experiments were conducted in a custom‐made chamber (35 × 35 × 35 cm^3^) with a square pillar (7 × 7 × 25 cm^3^) around which rats could move freely (Figure 1a). Prior to testing, animals were habituated to the chamber for 125 s. A ceiling‐mounted camera tracked the centroid of each rat's silhouette. The area around the pillar was virtually divided into eight fields, and auditory stimuli were assigned to fields and updated according to the animal's position. A ceiling‐mounted speaker (400‐SP082; Sanwa Direct) presented the assigned auditory stimuli. Acoustic calibration was performed with a 1/4‐in. microphone (Type 4939; Brüel & Kjaer, Nærum, Denmark) and a spectrum analyzer (CF‐5210; Ono Sokki Co. Ltd., Kanagawa, Japan) at the center of the chamber. The experiment consisted of multiple sessions and intervals. During the sessions, sound stimulus was presented from the speaker. Each session consisted of a 20‐s sound presentation followed by a 5‐s interval (Figure 1b). During the interval, 5 kHz pure tones were presented, and the field‐stimulus mapping was shuffled. Sound intensity levels are reported as dB SPL (sound pressure level) relative to 20 μPa. In Experiment 1, the two adjacent conditions were 0 dB SPL and 50 dB SPL white noise (25 sessions). In Experiment 2, four intensities (20/40/60/80 dB SPL) were arranged such that opposite diagonal fields shared the same intensity (50 sessions). Preferences were quantified by two metrics: (i) the length of stay, calculated as the cumulative time spent in each field, and (ii) the Preference Index (PI) (Soga et al. 2018). Because the length‐of‐stay metric can be confounded by freezing or immobility, the PI was calculated using the following equation.

$$\mathrm{PI}=\mathrm{Pr}\;\left(\text{Goal}=X/\text{Start}\neq X\right)-\mathrm{Pr}\;\left(\text{Goal}\neq X/\text{Start}=X\right)$$

where *Start = X* indicates that the rat was in position *X* at the beginning of the session, and *Goal = X* indicates that the rat was located in position *X* at the end of the session. *PI* focuses exclusively on movement, allowing us to exclude events where the rat stayed in one place due to place preference, freezing, sleep, motivational disengagement, or other forms of sustained immobility.

### 2.3 Surgery

**FIGURE 2:**
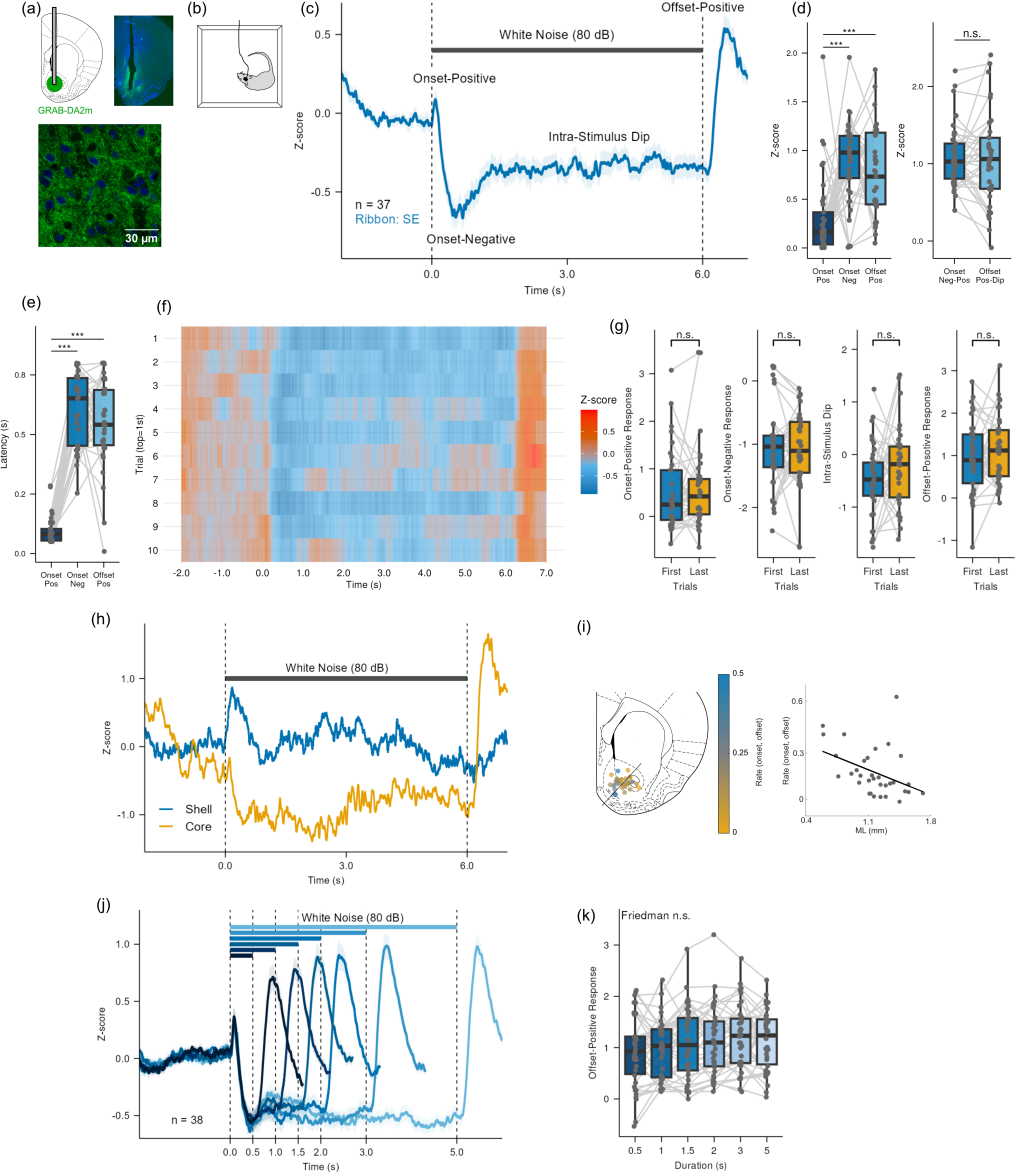
Characteristics of the dopamine response to white noise in the NAc. (a) Histological verification of recording sites. Top left: Schematic of viral injection (AAV‐hSyn‐GRAB‐DA2m) and fiber implantation targeting the NAc (AP 1.5–2.5 mm; ML 0.6–1.8 mm; DV 6.5–7.4 mm). Top right: Representative coronal brain section showing the implanted fiber track and GRAB‐DA2m expression (green) with DAPI nuclear counterstain (blue). Bottom: High‐magnification image of the NAc neurons expressing the sensor. (b) Experimental setup. Fiber photometry recordings were performed while rats moved freely in the chamber. (c) Average dopamine response (mean ± SEM; *n* = 37) to 6‐s, 80‐dB SPL white noise. The response comprised an onset‐positive peak, an onset‐negative trough, an intra‐stimulus dip, and an offset‐positive peak. The top bar indicates the stimulus duration. (d) Amplitude comparison from the same 6‐s trials, showing absolute amplitudes (left: *p* < 0.001) and relative amplitudes of the onset‐negative and offset‐positive components measured from their immediately preceding state (right; *p* = 0.531, *BF* = 0.179). Gray dots represent individual animals, and lines connect data from the same animal. (e) Peak latencies of the components shown in (c) (*p* < 0.001). (f) Trial‐by‐trial stability of the response in (c). Heatmap of grand‐average dopamine responses, with each row representing the across‐animal average for a single trial. (g) Comparisons of component amplitudes between the first and last trials for the onset‐positive, onset‐negative, intra‐stimulus dip, and offset‐positive components. No significant differences were observed (onset‐positive: *p* = 0.823, *BF* = 0.180; onset‐negative: *p* = 0.952, *BF* = 0.181; intra‐stimulus dip: *p* = 0.323, *BF* = 0.390; offset‐positive: *p* = 0.394, *BF* = 0.348). (h) Representative responses from the NAc shell (blue) and core (yellow). (i) Correlation between the onset‐positive/offset‐positive components ratio and recording location (*n* = 33). The ratio was calculated as: Ratio = onset‐positive / (onset‐positive + offset‐positive). Left: Reconstructed recording sites color‐coded by the ratio. The black line indicates a projection axis, defined from ventromedial (ML 0.0, DV 8.0) to dorsolateral (ML 8.0, DV 1.5) coordinates, running approximately orthogonal to the NAc shell‐core boundary. Right: The onset/offset ratio plotted against the projected position of each recording site along the axis, showing a significant negative correlation (Spearman's *r* = −0.4737, *p* = 0.00536). (j) Average responses to stimuli of varying durations (500–5000 ms; *n* = 38). Blue bars indicate stimulus durations. (k) Effect of duration on the offset‐positive component. No significant effect was observed (*p* = 0.140; *BF* = 1.526).

For fiber‐photometry experiments, a craniotomy was made over the NAc after horizontal alignment of the bregma and lambda. The dura was removed, and three additional small craniotomies were drilled to implant anchoring screws. A glass capillary was used to deliver AAV‐hSyn‐GRAB‐DA2m (Addgene#140553; total 500 nL) at 100 nL/min (Figure 2a; coordinates: AP 1.5–2.5 mm; ML 0.6–1.8 mm; DV 6.5–7.4 mm, relative to bregma) (Paxinos and Watson 2013). The capillary was left in place for 1–5 min before being slowly withdrawn to facilitate viral diffusion. An optical fiber cannula (400 μm core, NA 0.5, length 8–9 mm; Doric) was implanted 0.1 mm above the injection site and secured with dental adhesive and cement along with screws. Animals were allowed to recover for 2–3 weeks before the start of experiments.

### 2.4 Auditory Stimuli and Experimental Paradigms

We employed eight experimental paradigms, with some rats participating in more than one. For experiments with multiple conditions, the order of presentation was randomized. Experimenter blinding during data acquisition was not feasible because auditory stimuli and session progress had to be monitored in real time; however, signal processing and feature extraction were performed using prespecified, automated scripts to minimize bias. Unless otherwise noted, white noise at 80 dB SPL was presented 10 times with a 3‐s inter‐stimulus interval. The auditory stimuli were presented and calibrated using the same method as in the sound‐preference assay.

- Single presentation: continuous white noise for 6 s (*n* = 37).
- Multiple duration: white noise at durations of 500, 1000, 1500, 2000, 3000, or 5000 ms (*n* = 38).
- Dynamic‐change: 6‐s white noise with a level change at 3 s; initial 80 dB SPL stepping down to 80, 60, 40, or 20 dB SPL (*n* = 36).
- Constant‐change: same timing as above, with a fixed −40 dB SPL step; initial levels of 80, 70, 60, or 50 dB SPL (*n* = 36).
- Single brief presentation: 100‐ms white noise bursts for 100 times (*n* = 24).
- Repetitive presentation: 100‐ms bursts with a stimulus onset asynchrony (SOA) of 800, 600, 400, or 200 ms. A 2‐s pause was inserted after every 10 stimuli. Each condition comprised 1000 presentations (*n* = 36).
- Oddball: band‐limited noise (2250–2750 or 3250–3750 Hz; duration 150 ms; SOA 800 ms). One band served as standard and the other as deviant (10% probability), presented in pseudo‐random order with at least four consecutive standards (Figure 5a). After 300 deviants, roles of deviant and standard were swapped. To minimize temporal interaction, the standard for analysis was defined as the stimulus presented three trials before each deviant, providing a buffer and yielding a more stable measure (*n* = 21).
- Many‐standards control: the same rats from the oddball paradigm were presented with 10 band‐limited noises (1000–5500 Hz in 500‐Hz steps, 10% probability each). For analysis, the stimulus identical to the oddball deviant was labeled as the deviant‐equivalent.

Sample sizes were guided by a priori power analysis based on effect sizes reported in prior studies. Due to practical constraints inherent to long‐term fiber photometry experiments, some paradigms did not reach the initially recommended sample sizes. To compensate for the smaller *n* values, we focused on ensuring high within‐subject reliability and employed non‐parametric testing combined with Bayesian inference to verify the robustness of the observed effects.

### 2.5 Fiber Photometry

Extracellular dopamine dynamics in the NAc were measured optically (Doric system). During the recording, rats moved freely within the chamber (35 × 35 × 35 cm^3^) (Figure 2b). Excitation/isosbestic wavelength pairs were selected depending on the setup (475 nm/405 nm, 475 nm/440 nm, or 474 nm/405 nm). Each rat was recorded under a single wavelength configuration, and data were analyzed within‐animal to avoid cross‐setup confounds. In light of recent concerns about isosbestic referencing for GRAB sensors (Simpson et al. 2024), we adopted a conservative approach using the isosbestic channel as a reference. Raw fluorescence signals were processed offline. The isosbestic signal was linearly regressed onto the excitation signal (least squares method), and ΔF/F was calculated as follows:

$$\mathrm{\Delta F}/F=\left(F\left(\text{excitation}\right)-F\left(\text{isosbestic fitted}\right)\right)/F\left(\text{isosbestic fitted}\right).$$

To enable between‐condition comparisons and minimize photobleaching and baseline drift, signals were converted to z‐scores. For oddball and many‐standards control paradigms, z‐scores were computed within a −3200 to +3200 ms window around the deviant onset. This local normalization, rather than scaling against the entire recording, ensured that the deviant and corresponding standard responses are referenced to a shared local baseline, thereby minimizing the influence of slow drifts. For the other paradigms, z‐scores were computed across the entire recording session, and the baseline for visualization was defined as the 2‐s pre‐onset average. From each trace, we extracted representative features:

- Onset‐positive peak: maximum z‐score 1–300 ms after stimulus onset
- Onset‐negative trough: minimum z‐score 1–800 ms after onset
- Offset‐positive peak: maximum z‐score 1–800 ms after offset
- Intra‐stimulus dip: mean z‐score during the final 1 s of stimulus presentation

In short‐SOA paradigms (repetitive, oddball, many‐standards control), we also quantified the peak‐to‐trough difference within each SOA window.

### 2.6 Statistical Analysis

All analyses were performed within animals, treating each rat as its own control. Some animals were excluded from specific paradigms due to technical issues (e.g., equipment malfunction, unexpected death, or surgical failure), but no exclusions were based on experimental outcomes. Pairwise comparisons were performed using the Wilcoxon signed‐rank test and Bayesian paired‐samples *t*‐tests (Cauchy prior *r* = 0.707). Multi‐condition factors were analyzed using Friedman tests followed by Conover post hoc tests with Bonferroni correction, as well as Bayesian repeated‐measures ANOVA with multiple comparison correction by fixing the prior to 0.5 (Westfall et al. 1997). Bayes factors (*BF*) were interpreted as follows: *BF* > 3 indicated evidence for the alternative hypothesis, whereas *BF* < 0.33 indicated evidence for the null hypothesis (Kass and Raftery 1995). All analyses were conducted in JASP (JASP Team 2025).

### 2.7 Histology

After the recordings, the rats were anesthetized with an intraperitoneal injection of pentobarbital sodium (160 mg/kg) and then perfused transcardially with heparinized saline, followed by 4% paraformaldehyde (PFA) in 0.01 M phosphate‐buffered saline (PBS). The heads were post‐fixed in PFA overnight, after which the brains were removed and cryoprotected in 30% sucrose (0.1 M PBS). Coronal sections (50 μm) were cut using a Thermo Fisher cryostat, stained with DAPI, and examined to verify the cannula tip locations (Figure 2a).

## 3 Results

### 3.1 Preference for White Noise

To confirm that the white noise used in our experiments functioned as an aversive stimulus, we conducted a sound‐preference assay. Rats were allowed to move freely in the chamber, and auditory stimuli were delivered according to their position within eight virtual fields (Figure 1a,b). A representative trajectory is shown in Figure 1c. The cumulative heatmap of the rats' positions (Figure 1d) indicated no systematic place preferences across the arena. Even in trials with limited movement, the randomized reassignment of sound‐field mappings, together with the fact that the PI is based on field‐to‐field transitions, ensured that immobility did not bias the results. Preference was quantified using two measures: length of stay (the cumulative time spent in each field) and the PI.

In the 50 dB SPL versus silence comparison, rats showed a clear preference for silence (Figure 1e; length of stay: *p* < 0.001; PI: *p* < 0.001). Furthermore, when multiple sound intensities (20–80 dB SPL) were tested, preference decreased systematically as intensity increased (Figure 1f; Table S1; length of stay: *χ* ^2^(3) = 15.9, *p* < 0.001; PI: *χ* ^2^(3) = 21.4, *p* < 0.001). These findings are consistent with previous reports demonstrating the aversive nature of white noise in rats (Goedhoop et al. 2022; Soga et al. 2018).

### 3.2 Dopaminergic Responses to Sustained White Noise

Building on the finding that white noise is aversive, we next monitored dopamine dynamics in the NAc using fiber photometry in awake, freely moving rats. Recordings were conducted in the same custom chamber used for the behavioral assay (Figure 2b). During 6‐s white noise presentations, NAc dopamine exhibited a characteristic four‐phase response (Figure 2c). An onset‐positive peak occurred at 96 ± 9 ms (mean ± SEM; amplitude = 0.224 ± 0.075), followed by an onset‐negative trough at 602 ± 25 ms (amplitude = −0.860 ± 0.075). These were accompanied by an intra‐stimulus dip (amplitude = −0.327 ± 0.063). After sound offset, an offset‐positive peak was observed at 549 ± 30 ms (amplitude = 0.744 ± 0.080) before returning to baseline.

The absolute peak amplitudes of the onset‐positive, onset‐negative, and offset‐positive components differed significantly (Figure 2d; *p* < 0.001). Post hoc analysis revealed that the onset‐positive component differed significantly from both the onset‐negative and offset‐positive components, whereas no statistically significant difference was found between the onset‐negative and offset‐positive components (Table S2). Because the onset‐negative trough followed directly from the preceding onset‐positive peak, and the offset‐positive peak followed from the intra‐stimulus dip, we also quantified amplitude relative to the immediately preceding state. The relative amplitudes of the onset‐negative and offset‐positive components did not differ from each other (*p* = 0.531, *BF* = 0.179).

Peak latencies also differed across components (Figure 2e; *p* < 0.001). The onset‐positive latency differed from both the onset‐negative and offset‐positive latencies, whereas no significant difference was observed between the onset‐negative and offset‐positive latencies (Table S2).

To examine the stability of these responses, we further analyzed the 6‐s white noise trials on a trial‐by‐trial basis. Although NAc dopamine has been reported to attenuate following repeated stimulation (Legault and Wise 2001), we observed no such habituation. Trial‐averaged responses are shown as a heatmap in Figure 2f. A comparison of the first two and the last two trials revealed no significant differences in the onset‐positive, onset‐negative, intra‐stimulus dip, or offset‐positive components (Figure 2g; onset‐positive: *p* = 0.823, *BF* = 0.180; onset‐negative: *p* = 0.952, *BF* = 0.181; intra‐stimulus dip: *p* = 0.323, *BF* = 0.390; offset‐positive: *p* = 0.394, *BF* = 0.348).

Histological analysis revealed a topographic organization: the onset‐positive components were stronger in the NAc shell, whereas the offset‐positive components were stronger in the NAc core. The onset/offset ratio correlated with recording location (Figure 2h,i; Spearman's *r* = −0.4737, *p* = 0.00536). These findings are consistent with previous work showing that response dynamics depend on cannula placement (Bassareo et al. 2002; de Jong et al. 2019). Although these subregional differences were present, the results in the following sections include data from both subregions to provide a comprehensive overview of the dopamine response. Of the rats where histological verification was successfully completed, 18 had cannula tips located within the NAc core and 15 within the shell.

To examine the effect of movement on dopamine responses, we conducted an additional experiment with simultaneous motion tracking and dopamine recording (*n* = 11). We observed movements resembling avoidance behavior at stimulus onset. However, there was no significant correlation between movement velocity and either the onset‐positive or onset‐negative dopamine components (Figure S1; onset‐positive: *F* (1, 8.06) = 0.041, *p* = 0.844; onset‐negative: *F* (1, 22.59) = 1.367, *p* = 0.255). These findings indicate that the responses were triggered by the auditory stimulus rather than by movement.

### 3.3 Effect of Stimulus Duration

Having identified the multi‐phasic nature of the dopamine response, we next examined whether these components were modulated by stimulus duration. In the auditory cortex, the amplitude of offset responses has been reported to depend on tone burst duration (Takahashi et al. 2004). To determine whether a similar relationship exists in the NAc, we varied stimulus duration (500–5000 ms) and quantified the offset‐positive component. No significant effect of duration was observed (Figure 2j,k; *χ* ^2^(5) = 8.301, *p* = 0.140, *BF* = 1.526). Bayesian analysis indicated insufficient evidence to support either the null or the alternative hypothesis.

### 3.4 Effect of Intensity Change (Physical Salience)

Because stimulus duration did not significantly influence response magnitude, we examined whether NAc dopamine is sensitive to physical salience. To test this, we introduced mid‐stimulus intensity shifts using two paradigms: a dynamic‐change paradigm (an initial 80 dB SPL for 3 s followed by a shift to 80, 60, 40, or 20 dB SPL) and a constant‐change paradigm (initial intensities of 80, 70, 60, or 50 dB SPL followed by a fixed −40 dB drop at 3 s). Although the overall four‐phase pattern was preserved, we focused our analysis on dopamine dynamics during these intensity transitions.

**FIGURE 3:**
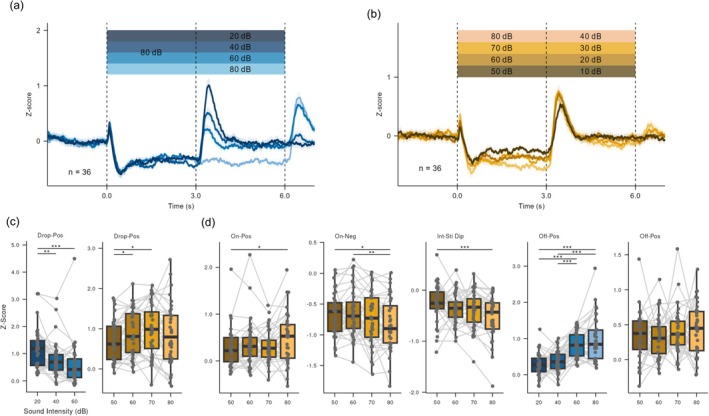
Dopamine responses track the magnitude of intensity change rather than absolute intensity levels. (a) Average dopamine responses (mean ± SEM; *n* = 36) in the dynamic‐change paradigm, in which stimulus intensity decreased at 3 s from a fixed 80 dB SPL. From dark to light colors, the second‐stage intensity was 20, 40, 60, or 80 dB SPL. (b) Average dopamine responses (mean ± SEM; *n* = 36) in the constant‐change paradigm, in which intensity was decreased by a fixed 40 dB SPL at 3 s from different initial levels. From dark to light colors, the initial intensities were 50, 60, 70, or 80 dB SPL. (c) Quantification of the drop‐positive responses. Left: Response from the dynamic‐change paradigm in (a) (*χ* ^2^(2) = 21.50, *p* < 0.001). Right: Responses from the dynamic‐change paradigm in (b) (*χ* ^2^(3) = 9.033, *p* = 0.029). (d) Quantification of responses of the onset‐positive, onset‐negative, intra‐stimulus dip, and offset‐positive. From left to right: onset‐positive (*χ* ^2^(3) = 7.233, *p* = 0.065), onset‐negative (*χ* ^2^(3) = 13.83, *p* = 0.003), intra‐stimulus dip (*χ* ^2^(3) = 15.77, *p* = 0.001), offset‐positive from (a) (*χ* ^2^(3) = 46.90, *p* < 0.001) and offset‐positive from (b) (*χ* ^2^(3) = 1.700, *p* = 0.637).

Notably, when the sound intensity decreased, we observed a positive deflection resembling the offset‐positive peak in both paradigms, which we termed the “drop‐positive” component (Figure 3a,b). In the dynamic‐change paradigm, the drop‐positive amplitude increased systematically with larger decrements (Figure 3c; Table S3; *χ* ^2^(2) = 21.50, *p* < 0.001). The 80‐to‐80 dB SPL condition was excluded from this specific analysis because it involved no actual intensity change. In the constant‐change paradigm, drop‐positive components differed across initial intensities (Figure 3c; Table S3; *χ* ^2^(3) = 9.033, *p* = 0.029). Post hoc analysis revealed that this effect was driven by the 50‐dB SPL condition, specifically the 70/50 and 60/50 dB SPL comparisons. All other pairs were statistically indistinguishable, a result supported by Bayesian analysis (Table S3). This suggests that the response does not track the starting intensity, except when the post‐change level drops to a near‐inaudible threshold. Taken together, these findings indicate that drop‐positive components encode the magnitude of the intensity change rather than the absolute pre‐ or post‐change intensity levels.

The remaining components of the dopamine response also exhibited differential sensitivity to intensity. The onset‐positive component showed a trend across conditions, with a significant difference detected only in the 80/50 dB SPL comparison (Figure 3d; Table S4; *χ* ^2^(3) = 7.233, *p* = 0.065). In contrast, both the onset‐negative component (*χ* ^2^(3) = 13.83, *p* = 0.003) and the intra‐stimulus dip (*χ* ^2^(3) = 15.77, *p* = 0.001) differed significantly across conditions, indicating higher sensitivity to physical salience (Figure 3d; Table S4). For the offset‐positive component, its magnitude reflected sound intensity only within the audible range. In the dynamic‐change paradigm, the offset‐positive component scaled with the second‐stage intensity (Figure 3d; Table S4; *χ* ^2^(3) = 46.90, *p* < 0.001), whereas this relationship was absent in the constant‐change paradigm (Figure 3d; *χ* ^2^(3) = 1.700, *p* = 0.637, *BF* = 0.099). Collectively, these findings indicate that dopaminergic modulation is driven primarily by intensity differences rather than absolute intensity levels.

### 3.5 Responses to Brief Stimuli

**FIGURE 4:**
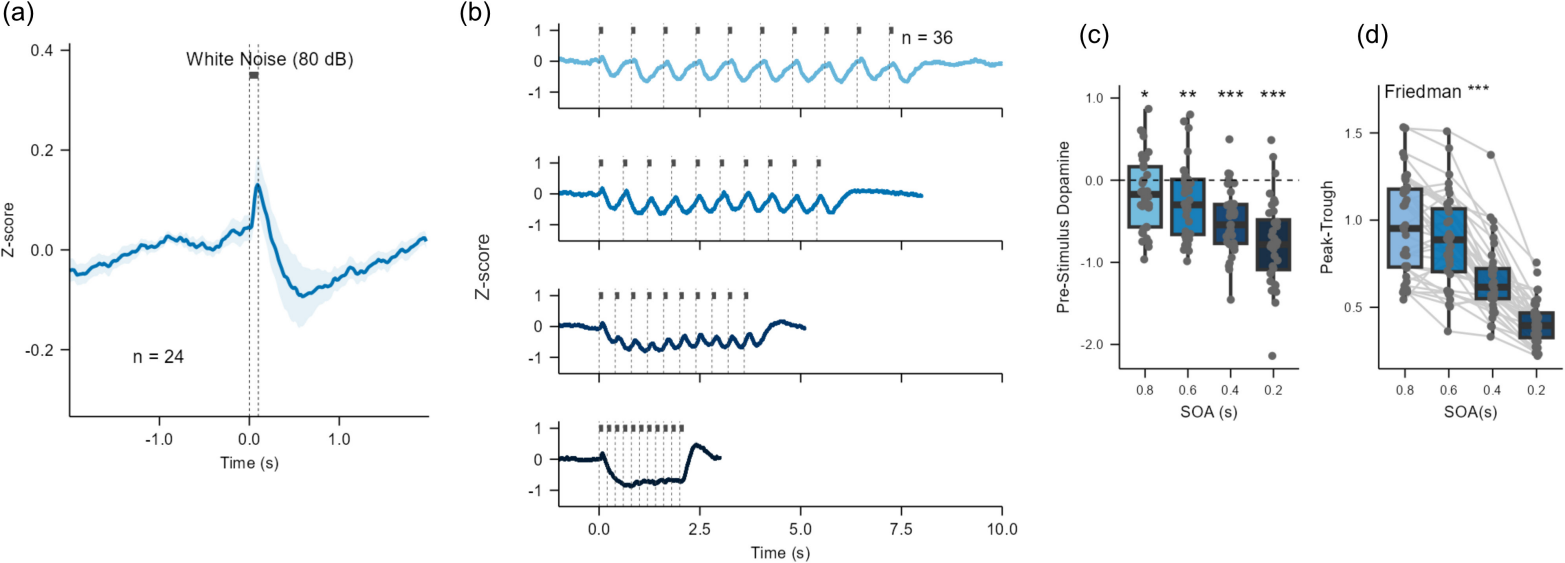
Dopamine responses to brief and repetitive stimuli. (a) Average dopamine response to a single 100‐ms white noise burst (mean ± SEM, *n* = 24). (b) Average dopamine response to 100‐ms bursts presented with SOAs of 800, 600, 400, and 200 ms (top to bottom; *n* = 36). (c) Dopamine level (z‐score) at stimulus onset for each SOA condition, showing incomplete recovery at shorter SOAs. (d) Peak‐to‐trough amplitude differences for each SOA condition, showing response attenuation at shorter SOAs (*χ* ^2^(3) = 97.033, *p* < 0.001).

To characterize the temporal dynamics of dopamine responses to auditory stimuli, we presented brief white noise bursts. A 100‐ms white noise burst elicited a component profile similar to longer stimuli: an onset‐positive peak at 118 ± 14 ms, followed by an onset‐negative trough at 502 ± 37 ms, and a subsequent recovery toward baseline (Figure 4a). Notably, the onset‐negative component was observed even after stimulus offset. With shorter SOAs (800, 600, 400, and 200 ms), clear oscillatory responses were evident in the 800–400 ms conditions but largely merged into an intra‐stimulus dip at 200 ms (Figure 4b). Baseline levels at stimulus onset remained displaced, reflecting incomplete recovery from the prior trial (Figure 4c; 800 ms: *p* = 0.043; 600 ms: *p* = 0.002; 400 ms: *p* < 0.001; 200 ms: *p* < 0.001). The peak‐to‐trough difference within each SOA window differed significantly across conditions (Figure 4d; Table S5, *χ* ^2^(3) = 97.033, *p* < 0.001).

### 3.6 Oddball Versus Many‐Standards Control

**FIGURE 5:**
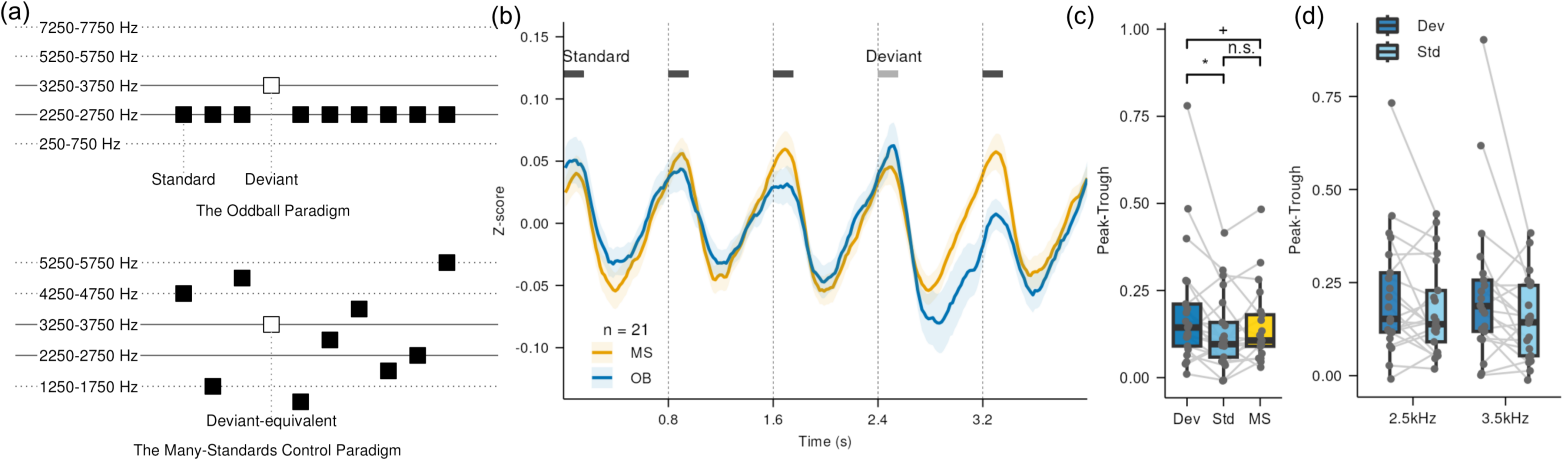
Dopamine responses to the oddball and many‐standards control paradigms. (a) Schematic of the auditory stimuli. The vertical axis schematically represents different stimulus frequencies. Top: In the oddball paradigm, a rare deviant stimulus (white square) interrupts a sequence of repeating standard stimuli (black squares). Bottom: In the many‐standards control paradigm, 10 different band‐limited noises (black squares) are presented with equal probability (10% each), so no single stimulus forms a repeating standard. The “deviant‐equivalent” (white square) is physically identical to the deviant in the oddball paradigm. (b) Average dopamine response (mean ± SEM; *n* = 21) aligned to the onset of the deviant (oddball paradigm, OB, blue) or deviant‐equivalent (many‐standards control paradigm, MS, yellow) stimulus. Dotted lines indicate stimulus onsets. (c) Peak‐to‐trough amplitude for the deviant (Dev) and standard (Std) stimuli in the oddball paradigm, and the deviant‐equivalent stimulus in the many‐standards control paradigm (MS) (deviant‐standard: *p* = 0.011; deviant‐“deviant‐equivalent”: *p* = 0.089; standard‐“deviant‐equivalent”: *p* = 0.216; *BF* = 0.417). (d) Average dopamine responses to deviants at two frequencies (2.5 and 3.5 kHz) and their corresponding standards. Dark blue and light blue represent deviant (Dev) and standard (Std) stimuli, respectively. Two‐way ANOVA revealed a significant main effect of the stimulus role (Dev vs. Std; *p* = 0.041), with no effect of frequency (*p* = 0.649) and no interaction (*p* = 0.190).

Building on the observed sensitivity to temporal transitions and intensity contrasts, we finally examined whether these dopaminergic modulations also reflect violations of statistical regularity. To address this question, we employed 150‐ms band‐limited noise with an 800‐ms SOA for the oddball and many‐standards control paradigm. The stimulus duration was extended from 100 to 150 ms to improve detection of onset components. Because the 800‐ms SOA did not allow full recovery of the dopamine signal, consecutive responses were temporally dependent (Figure 4c). To ensure that analyses compared temporally independent events, the standard response was defined as the stimulus presented three trials before each deviant, providing a sufficient buffer and a stable reference of the standard response. Regardless of stimulus type, band‐limited noise elicited a stimulus‐locked waveform in the dopamine signal (Figure 5b). Because a stable baseline could not be defined under continuous stimulation, responses were quantified as the peak‐to‐trough amplitude difference within the SOA window.

In the oddball paradigm, responses to deviants were significantly larger than those to standards (Figure 5c; *p* = 0.011). To assess potential frequency‐specific effects, we analyzed responses separately for 2.5‐ and 3.5‐kHz deviants. A two‐way ANOVA revealed no significant interaction between sound frequency and stimulus condition (deviant vs. standard; *p* = 0.190) and no main effect of frequency (*p* = 0.649), but a significant main effect of stimulus condition (Figure 5d; *p* = 0.041). These findings indicate that the enhanced dopamine response to deviants was consistent across both frequency bands, with no evidence of frequency‐dependent modulation.

For our core comparison, we contrasted the oddball deviant with the physically identical ‘deviant‐equivalent’ stimulus in the many‐standards control paradigm. The response tended to be larger in the oddball context (Figure 5c; *p* = 0.089). By contrast, no significant difference was found between the deviant‐equivalent and the standard (Figure 5c; *p* = 0.216; *BF* = 0.417). Taken together, these findings demonstrate that NAc dopamine responses are selectively enhanced not by mere rarity or adaptation, but by violations of statistical regularity.

## 4 Discussion

This study tested the hypothesis that phasic dopamine activity in the NAc signals the salience of violations of ongoing statistical regularities. Our key findings demonstrate that (i) sustained auditory stimuli elicit a multi‐component dopamine response that can be functionally parsed into alerting, value‐related, and relief components; (ii) the dopamine response is significantly amplified for stimuli that violate a statistical rule, but not for physically identical stimuli that are merely rare; and (iii) the latency of this deviance‐related signal (~500 ms) is substantially longer than that of the cortical MMN. Taken together, these results support our hypothesis that NAc dopamine encodes salience specifically tied to rule violation and are more consistent with a downstream precision broadcast than with an upstream non‐parallel broadcast, in which dopamine would be expected to precede and directly drive cortical deviance signals. By bridging the historically distinct fields of predictive coding and reinforcement learning, our findings position NAc dopamine as a key downstream modulator that evaluates and weights cortically detected deviance to guide adaptive behavior.

### 4.1 A Multi‐Component Dopamine Response to Auditory Stimuli

We identified four distinct components in the dopamine response to auditory stimuli: an initial positive peak at stimulus onset (onset‐positive), a subsequent negative peak (onset‐negative), a sustained decrease during stimulus presentation (intra‐stimulus dip), and a final positive peak after stimulus offset (offset‐positive). Multi‐phasic dopamine responses have been previously interpreted in terms of alerting and value‐related functional components. Our findings are broadly consistent with this framework.

The initial onset‐positive component exhibits features consistent with an alerting response, as it was observed across diverse sounds and scaled with stimulus intensity (Bromberg‐Martin et al. 2010; Goedhoop et al. 2022; Gonzalez et al. 2023). Notably, unlike the attenuation typically observed for novel, non‐aversive stimuli (Legault and Wise 2001), this response remained stable across trials, suggesting that the white noise retained its behavioral significance throughout repetition.

Within the established framework of dopamine signaling, the component that follows a rapid alerting signal is typically associated with stimulus value encoding (Berke 2018; Ljungberg et al. 1992; Schultz 2016). In our data, this framework suggests that the onset‐negative component and intra‐stimulus dip reflect value‐related processing. Their negative polarity is consistent with behavioral evidence indicating that white noise is aversive to rats. In this context, the offset‐positive and drop‐positive components may represent signals of relief from aversion (Harris and Peng 2020; Mayer et al. 2018; Moutoussis et al. 2008). Moreover, the latency and amplitude of the onset‐negative and offset‐positive components were comparable, suggesting a symmetrical role in value encoding. Intriguingly, the ratio of the onset‐positive to the offset‐positive components correlated with the anatomical location along the NAc core‐shell axis. Although this finding hints at a functional topography of dopamine dynamics, both subregions exhibited the same fundamental multi‐phasic response pattern.

A critical insight into these dynamics comes from our intensity‐transition experiments. We found that both the onset‐negative (intensity increase) and drop‐positive (intensity decrease) components scaled with the magnitude of the intensity transition. Importantly, the drop‐positive component tracked the magnitude of the change rather than the absolute sound level. This pattern suggests that the NAc dopamine does not simply mirror sensory input, but instead reflects a processed signal related to the salience and evaluated significance of environmental changes.

### 4.2 Rule Violation, Not Rarity, Drives Enhanced Dopamine Signaling

Our paradigm isolated the violation of an ongoing statistical regularity by contrasting the oddball paradigm with the many‐standards control paradigm. The dopamine response was greater for deviant stimuli, allowing us to refine the broader concept of salience. Salience is known to comprise multiple dimensions, including physical properties, motivational value, and novelty (Bromberg‐Martin et al. 2010; Horvitz 2000; Schultz 2013, 2016). Our results provide direct evidence that it also includes empirical salience: significance derived not from a stimulus's intrinsic properties but from its violation of a statistical context. This interpretation is further supported by the finding that the response to the rare deviant‐equivalent stimulus was indistinguishable from that to the common standard stimulus, thereby isolating rule violation as the critical factor driving the enhanced dopamine signal. Furthermore, our analyses revealed no evidence that the deviant‐related dopamine signal was influenced by the physical frequency of the sound.

Although it is challenging to isolate distinct response components in the oddball paradigm, the enhanced response to deviants appears to be primarily driven by the value‐encoding component, as a more pronounced decrease in dopamine was observed. This pattern suggests that deviant stimuli may be perceived as more aversive than standard stimuli. Given that unpredictable stimuli are often experienced as aversive (Goedhoop et al. 2022; Schaap et al. 2013), one possible interpretation is that the enhanced dopamine response reflects not the salience of deviance per se, but a heightened negativity associated with unpredictability. However, if the response were solely a reflection of negative valence due to low stimulus probability, a similar enhancement should have been observed in the many‐standards control paradigm. Instead, the dopamine response was smaller in the many‐standards control paradigm than in the oddball paradigm. This key finding dissociates the salience of the prediction error from mere negativity arising from rareness.

### 4.3 Deviance, Salience, and Precision: A Combined Model

Our findings clarify that the observed NAc dopamine signal does not represent deviance detection itself, but rather a distinct process of salience evaluation. Several lines of evidence support this interpretation. First, the peak latency of the deviance‐related dopamine response was approximately 500 ms, substantially longer than that of the cortical MMN—the canonical marker of initial deviance detection (Carbajal and Malmierca 2018; Fitzgerald and Todd 2020; Shiramatsu et al. 2013; Shiramatsu and Takahashi 2021). Second, NAc dopamine responded even to standard stimuli, in contrast to the stimulus‐specific adaptation observed in the auditory cortex (Carbajal and Malmierca 2018; Fitzgerald and Todd 2020; Näätänen et al. 2007). Finally, these functional and temporal distinctions align with pharmacological evidence showing that dopamine manipulation does not alter the MMN (Inaba et al. 2021; Leung et al. 2007). Together, these findings argue against the direct role of NAc dopamine in generating cortical prediction errors. Instead, the dopamine response is better characterized as a salience signal, consistent with the broader view that dopamine encodes the general salience of events, extending beyond reward prediction error (Bromberg‐Martin et al. 2010; Gonzalez et al. 2023; Kutlu et al. 2021; Schultz 2013, 2016).

We can further refine this concept by positioning the dopamine salience signal within a parallel processing model grounded in the predictive coding framework (Berke 2018; Friston et al. 2012, 2015). In this model, a deviant event triggers two distinct computations. In the cortex, a prediction error signal is computed. Crucially, this signal is not a raw sensory mismatch but is already weighted by the stimulus's learned “empirical salience,” reflecting its statistical rarity and behavioral relevance (Shiramatsu and Takahashi 2018). We denote this empirically weighted prediction error as δ'(t). In parallel, the mesolimbic dopamine system evaluates the motivational significance of the same event and broadcasts a separate “precision term,” π(t). Functionally, this precision term acts as a gain factor on the cortical prediction error, producing an effective teaching signal, π(t)·δ'(t), that governs the allocation of attention and the degree of learning. This proposed parallel model provides a compelling framework for interpreting our two key findings: (i) the delayed peak of the dopamine response relative to the MMN, and (ii) its selective enhancement for rule violation over mere rarity. Both features are consistent with a model in which two distinct, but functionally integrated signals are generated in parallel to guide adaptive behavior.

### 4.4 Methodological Considerations and Future Directions

We used fiber photometry in freely moving rats, a technique that provides neurotransmitter specificity but requires careful interpretation. A primary limitation of our study is that we did not simultaneously record the cortical MMN and striatal dopamine signals; our interpretation relies on comparing latencies reported in separate studies. Although such cross‐study comparisons must be approached with caution, the genetically encoded sensor used here (GRAB‐DA2m) exhibits sub‐second kinetics with a rise time of approximately 70 ms (Sun et al. 2020), enabling detection of rapid dopamine transients following auditory deviance. Accordingly, the observed latency difference is consistent with a temporal delay between cortical deviance detection and the subsequent dopamine response, rather than being solely attributable to sensor kinetics. Future experiments incorporating simultaneous recordings will be essential to test directed interactions between these regions. For example, such studies could determine whether the amplitude of the cortical MMN predicts the magnitude of the subsequent NAc dopamine response, or whether pre‐stimulus dopamine levels modulate the amplitude and plasticity of the MMN. These next steps will be crucial for clarifying the precise causal relationships between cortical prediction error and subcortical salience signaling. In addition, manipulating the motivational context will help to dissociate empirical salience (arising from statistical violation) more clearly from motivational salience (arising from value or reinforcement). Future studies could also leverage optogenetic or chemogenetic tools to manipulate dopaminergic activity with millisecond precision, providing causal tests of whether NAc dopamine functions as a downstream precision broadcast or as an upstream driver of cortical deviance signals.

## 5 Conclusion

This study provides evidence that NAc dopamine encodes the salience of violations of ongoing statistical regularities, independent of reward. By showing that this signal reflects the cognitive significance of a prediction error rather than mere sensory features, and that it emerges substantially later than the cortical mismatch signal, our findings help to bridge the historically separated fields of predictive coding and reinforcement learning. We propose that NAc dopamine functions as a parallel precision broadcast—a mechanism that informs downstream circuits about the significance of unexpected events and modulates the impact of cortically detected prediction errors. These results clarify a fundamental process by which the brain prioritizes unexpected events to adaptively update its internal model of a dynamic world.

## Author Contributions

**Riko Iizuka:** conceptualization (equal), data curation (lead), formal analysis (lead), investigation (equal), methodology (equal), software (lead), visualization (lead), writing – original draft (lead), writing – review and editing (equal). **Ryotaro Yamaki:** conceptualization (equal), formal analysis (equal), investigation (equal), methodology (equal), software (equal), writing – original draft (supporting), writing – review and editing (equal). **Tomoyo Isoguchi Shiramatsu:** conceptualization (supporting), funding acquisition (equal), methodology (supporting), project administration (equal), resources (equal), supervision (equal), writing – review and editing (equal). **Shota Morikawa:** methodology (supporting), resources (equal), supervision (equal), writing – review and editing (equal). **Yuji Ikegaya:** funding acquisition (equal), methodology (supporting), resources (equal), supervision (equal), writing – review and editing (equal). **Hirokazu Takahashi:** conceptualization (supporting), funding acquisition (equal), methodology (supporting), project administration (lead), resources (equal), supervision (equal), writing – original draft (supporting), writing – review and editing (lead).

## Funding

This work was supported by JSPS KAKENHI (23H03465, 23H04336, 23H03023, 24H01544, 25KJ1020, 25H02600, 25K22825), AMED (JP23dm0307009, 24wm0625401h0001), JST (JPMJPR22S8, JPMJSP2108), the Asahi Glass Foundation, and the Secom Science and Technology Foundation.

## Conflicts of Interest

The authors declare no conflicts of interest.

## Supporting information


**Table S1:** Conover's post hoc comparisons for behavioral assessment of aversion to white noise between 20 dB SPL to 80 dB SPL. (a) Length of stay. (b) PI.
**Table S2:** Post hoc comparisons for amplitudes and latencies of multiple components. (a) Conover's post hoc comparison for amplitudes. (b) Bayesian ANOVA post hoc comparison for amplitudes. (c) Conover's post hoc comparison for latencies.
**Table S3:** Conover's post hoc comparisons for the drop‐positive components. (a) Dynamic‐change paradigm. (b) Constant‐change paradigm. (c) Bayesian ANOVA post hoc comparison for constant‐change paradigm.
**Table S4:** Conover's post hoc comparisons for components. (a) Onset‐positive, constant‐change paradigm. (b) Onset‐negative, constant‐change paradigm. (c) Intra‐stimulus dip, constant‐change paradigm. (d) Offset‐positive, dynamic‐change paradigm.
**Table S5:** Conover's post hoc comparisons for the peak‐to‐trough difference for brief white noise bursts.
**Figure S1:** Simultaneous recordings of motion and dopamine. (a) Heatmaps showing motion speed (left) and dopamine response (right) aligned to the white noise onset (dashed line, 0 s) across 200 consecutive trials. Motion was tracked at 30 fps via DeepLabCut (Mathis et al. 2018) and smoothed with a 3‐point mean filter. (b, c) Relationship between motion speed and dopamine response amplitudes. Scatter plots display the mean motion speed plotted against the amplitude of the onset‐positive component (b) and the onset‐negative component (c). Mean motion speeds were calculated within the specific time windows used to detect each component: 1–300 ms for the onset‐positive component, 1–800 ms for the onset‐negative component. Colors represent individual animals. A linear mixed‐effects model was used to account for the hierarchical nature of the data (trials nested within animals). No significant correlation was found for either the onset‐positive component (*F* (1, 8.06) = 0.041, *p* = 0.844) or the onset‐negative component (*F* (1, 22.59) = 1.37, *p* = 0.255).

## Data Availability

The data that support the findings of this study are openly available in Zenodo at https://doi.org/10.5281/zenodo.17221333, reference number 17221333.
