# Supplementary material for: Dopamine in the Nucleus Accumbens Signals Salience of Auditory Deviance
Source: Eur J Neurosci. 2026 Apr 2;63(7):e70486. doi: 10.1111/ejn.70486 (PMC13044869; doi:10.1111/ejn.70486)
Supplement: Supplementary file 1 — Table S1: Conover's post hoc comparisons for behavioral assessment of aversion to white noise between 20 dB SPL to 80 dB SPL. (a) Length of stay. (b) PI. Table S2: Post hoc comparisons for amplitudes and latencies of multiple components. (a) Conover's post hoc comparison for amplitudes. (b) Bayesian ANOVA post hoc comparison for amplitudes. (c) Conover's post hoc comparison for latencies. Table S3: Conover's post hoc comparisons for the drop‐positive components. (a) Dynamic‐change paradigm. (b) Constant‐change paradigm. (c) Bayesian ANOVA post hoc comparison for constant‐change paradigm. Table S4: Conover's post hoc comparisons for components. (a) Onset‐positive, constant‐change paradigm. (b) Onset‐negative, constant‐change paradigm. (c) Intra‐stimulus dip, constant‐change paradigm. (d) Offset‐positive, dynamic‐change paradigm. Table S5: Conover's post hoc comparisons for the peak‐to‐trough difference for brief white noise bursts. Figure S1: Simultaneous recordings of motion and dopamine. (a) Heatmaps showing motion speed (left) and dopamine response (right) aligned to the white noise onset (dashed line, 0 s) across 200 consecutive trials. Motion was tracked at 30 fps via DeepLabCut (Mathis et al. 2018) and smoothed with a 3‐point mean filter. (b, c) Relationship between motion speed and dopamine response amplitudes. Scatter plots display the mean motion speed plotted against the amplitude of the onset‐positive component (b) and the onset‐negative component (c). Mean motion speeds were calculated within the specific time windows used to detect each component: 1–300 ms for the onset‐positive component, 1–800 ms for the onset‐negative component. Colors represent individual animals. A linear mixed‐effects model was used to account for the hierarchical nature of the data (trials nested within animals). No significant correlation was found for either the onset‐positive component (F (1, 8.06) = 0.041, p = 0.844) or the onset‐negative component (F (1, 22.59) = 1.37, p = 0. [file EJN-63-0-s001.pdf]

Table S1

Conover's post hoc comparisons for behavioral assessment of aversion to white noise between 20 dB SPL to 80 dB SPL. (a) Length of Stay (b) PI

(a)

Conover's Post Hoc Comparisons – Length of Stay

		T-Stat	p	p_{bonf}
20 dB SPL	40 dB SPL	2.853	0.007	0.045
	60 dB SPL	4.754	< .001	< .001
	80 dB SPL	6.656	< .001	< .001
40 dB SPL	60 dB SPL	1.902	0.066	0.396
	80 dB SPL	3.803	< .001	0.004
60 dB SPL	80 dB SPL	1.902	0.066	0.396

Note. Grouped by subject.

Note. Rank-biserial correlation based on individual signed-rank tests.

(b)

Conover's Post Hoc Comparisons - PI

		T-Stat	p	p_{bonf}
20 dB SPL	40 dB SPL	1.418	0.166	0.993
	60 dB SPL	3.849	< .001	0.003
	80 dB SPL	4.457	< .001	< .001
40 dB SPL	60 dB SPL	2.431	0.021	0.124
	80 dB SPL	3.039	0.005	0.028
60 dB SPL	80 dB SPL	0.608	0.547	1

Note. Grouped by subject.

Note. Rank-biserial correlation based on individual signed-rank tests.

Table S2

Post hoc comparisons for amplitudes and latencies of multiple components. (a) Conover's post hoc comparison for amplitudes (b) Bayesian ANOVA post hoc comparison for amplitudes (c) Conover's post hoc comparison for latencies

(a) *Conover's Post Hoc Comparisons - amplitudes*

		T-Stat	p	p_{bonf}
Onset-Positive	Onset-Negative	6.007	< .001	< .001
	Offset-Positive	4.72	< .001	< .001
Onset-Negative	Offset-Positive	1.287	0.202	0.606

Note. Grouped by subject.

Note. Rank-biserial correlation based on individual signed-rank tests.

(b) *Bayesian ANOVA Post Hoc Comparisons - amplitudes*

		Posterior Odds	$BF_{10, U}$	error %
Onset-Positive	Onset-Negative	475.294	809.147	1.986×10^{-6}
	Offset-Positive	746.929	1271.583	3.309×10^{-6}
Onset-Negative	Offset-Positive	0.195	0.331	0.038

Note. The posterior odds have been corrected for multiple testing by fixing to 0.5 the prior probability that the null hypothesis holds across all comparisons (Westfall, Johnson, & Utts, 1997). Individual comparisons are based on the default t-test with a Cauchy (0, r = 1/sqrt(2)) prior. The "U" in the Bayes factor denotes that it is uncorrected.

(c) *Conover's Post Hoc Comparisons - latencies*

		T-Stat	p	p_{bonf}
Onset-Positive	Onset-Negative	12.277	< .001	< .001
	Offset-Positive	10.985	< .001	< .001
Onset-Negative	Offset-Positive	1.292	0.2	0.601

Note. Grouped by subject.

Note. Rank-biserial correlation based on individual signed-rank tests.

(d) *Bayesian ANOVA Post Hoc Comparisons - latencies*

		Posterior Odds	$BF_{10, U}$	error %
Onset-Positive	Onset-Negative	$9.829\times10^{+17}$	$1.673\times10^{+18}$	2.692×10^{-20}
	Offset-Positive	$7.726\times10^{+12}$	$1.315\times10^{+13}$	2.984×10^{-16}
Onset-Negative	Offset-Positive	0.29	0.493	0.033

Note. The posterior odds have been corrected for multiple testing by fixing to 0.5 the prior probability that the null hypothesis holds across all comparisons (Westfall, Johnson, & Utts, 1997). Individual comparisons are based on the default t-test with a Cauchy (0, r = 1/sqrt(2)) prior. The "U" in the Bayes factor denotes that it is uncorrected.

Table S3

Conover's post hoc comparisons for the drop-positive components. (a) dynamic-change paradigm (b) constant-change paradigm. (c) Bayesian ANOVA post hoc comparison for constant-change paradigm.

(a) *Conover's Post Hoc Comparisons -dynamic change, drop positive*

		T-Stat	p	p_{bonf}
60 dB SPL	40 dB SPL	2.081	0.041	0.123
	20 dB SPL	5.411	< .001	< .001
40 dB SPL	20 dB SPL	3.33	0.001	0.004

Note. Grouped by subject.

Note. Rank-biserial correlation based on individual signed-rank tests.

(b) *Conover's Post Hoc Comparisons - constant change, drop positive*

		T-Stat	p	p_{bonf}
80 dB SPL	70 dB SPL	2.351	0.021	0.124
	60 dB SPL	1.41	0.161	0.968
	50 dB SPL	0.376	0.708	1
70 dB SPL	60 dB SPL	0.94	0.349	1
	50 dB SPL	2.727	0.007	0.045
60 dB SPL	50 dB SPL	1.787	0.077	0.461

Note. Grouped by subject.

Note. Rank-biserial correlation based on individual signed-rank tests.

(c) *Bayesian ANOVA Post Hoc Comparisons - constant change, drop positive*

		Posterior Odds	$BF_{10, U}$	error %
80 dB SPL	70 dB SPL	0.217	0.524	0.031
	60 dB SPL	0.081	0.196	0.044
	50 dB SPL	0.165	0.399	0.035
70 dB SPL	60 dB SPL	0.141	0.341	0.037
	50 dB SPL	5.793	13.985	6.644×10^{-8}
60 dB SPL	50 dB SPL	3.429	8.279	1.802×10^{-7}

Note. The posterior odds have been corrected for multiple testing by fixing to 0.5 the prior probability that the null hypothesis holds across all comparisons (Westfall, Johnson, & Utts, 1997). Individual comparisons are based on the default t-test with a Cauchy (0, r = 1/sqrt(2)) prior. The "U" in the Bayes factor denotes that it is uncorrected.

Table S4

Conover's Post Hoc Comparisons for components. (a) Onset-Positive, constant-change paradigm (b) Onset-Negative, constant-change paradigm (c) Intra-Stimulus Dip, constant-change paradigm (d) Offset-Positive, dynamic-change paradigm

(a)

Conover's Post Hoc Comparisons - constant change, onset positive

		T-Stat	p	p_{bonf}
80 dB SPL	70 dB SPL	1.771	0.08	0.477
	60 dB SPL	1.491	0.139	0.834
	50 dB SPL	2.702	0.008	0.048
70 dB SPL	60 dB SPL	0.28	0.78	1
	50 dB SPL	0.932	0.354	1
60 dB SPL	50 dB SPL	1.211	0.228	1

Note. Grouped by subject.

Note. Rank-biserial correlation based on individual signed-rank tests.

(b)

Conover's Post Hoc Comparisons - constant change, onset negative

		T-Stat	p	p_{bonf}
80 dB SPL	70 dB SPL	1.832	0.07	0.419
	60 dB SPL	3.663	< .001	0.002
	50 dB SPL	2.988	0.003	0.021
70 dB SPL	60 dB SPL	1.832	0.07	0.419
	50 dB SPL	1.157	0.25	1
60 dB SPL	50 dB SPL	0.675	0.501	1

Note. Grouped by subject.

Note. Rank-biserial correlation based on individual signed-rank tests.

(c)

Conover's Post Hoc Comparisons - constant change, intra stimulus dip

		T-Stat	p	p_{bonf}
80 dB SPL	70 dB SPL	2.63	0.01	0.059
	60 dB SPL	2.143	0.034	0.207
	50 dB SPL	4.188	< .001	< .001
70 dB SPL	60 dB SPL	0.487	0.627	1
	50 dB SPL	1.558	0.122	0.733
60 dB SPL	50 dB SPL	2.045	0.043	0.26

Note. Grouped by subject.

Note. Rank-biserial correlation based on individual signed-rank tests.

(d)

Conover's Post Hoc Comparisons - dynamic change, offset positive

		T-Stat	p	p_{bonf}
80 dB SPL	60 dB SPL	0.12	0.905	1
	40 dB SPL	6.582	< .001	< .001
	20 dB SPL	6.223	< .001	< .001
60 dB SPL	40 dB SPL	6.462	< .001	< .001
	20 dB SPL	6.103	< .001	< .001
40 dB SPL	20 dB SPL	0.359	0.72	1

Note. Grouped by subject.

Note. Rank-biserial correlation based on individual signed-rank tests.

Table S5

Conover's post hoc comparisons for the peak-to-trough difference for brief white noise bursts.

Conover's Post Hoc Comparisons - SOA condition

		T-Stat	p	p_{bonf}
0.8 s	0.6 s	5.649	< .001	< .001
	0.4 s	17.23	< .001	< .001
	0.2 s	27.964	< .001	< .001
0.6 s	0.4 s	11.581	< .001	< .001
	0.2 s	22.315	< .001	< .001
0.4 s	0.2 s	10.734	< .001	< .001

Note. Grouped by subject.

Note. Rank-biserial correlation based on individual signed-rank tests.

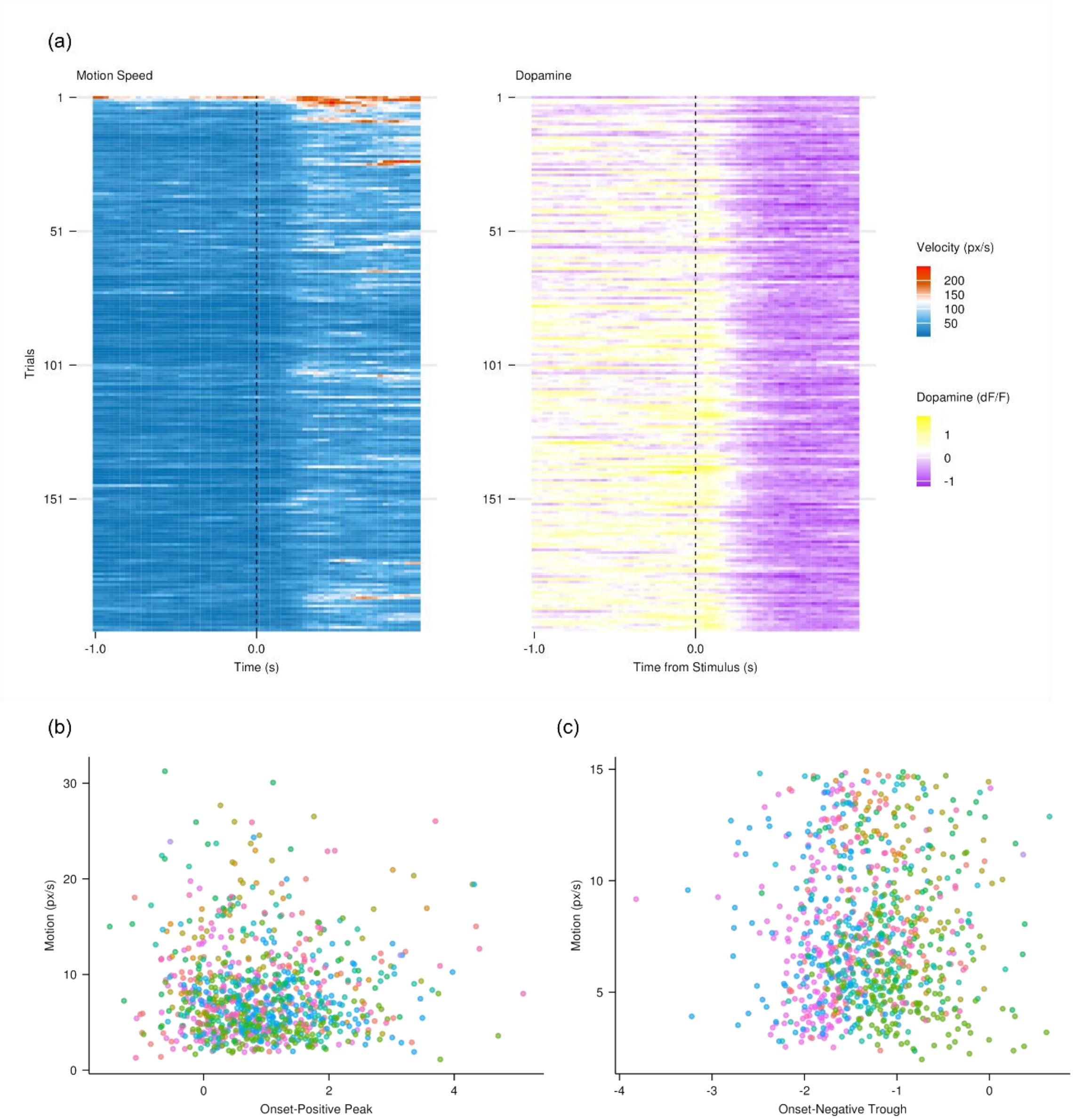

Figure S1

Simultaneous recordings of motion and dopamine. (a) Heatmaps showing motion speed (left) and dopamine response (right) aligned to the white noise onset (dashed line, 0 s) across 200 consecutive trials. Motion was tracked at 30 fps via DeepLabCut (Mathis et al., 2018) and smoothed with a 3-point mean filter. (b, c) Relationship between motion speed and dopamine response amplitudes. Scatter plots display the mean motion speed plotted against the amplitude of the onset-positive component (b) and the onset-negative component (c). Mean motion speeds were calculated within the specific time windows used to detect each component: 1–300 ms for the onset-positive component, 1–800 ms for the onset-negative component. Colors represent individual animals. A linear mixed-effects model was used to account for the hierarchical nature of the data (trials nested within

animals). No significant correlation was found for either the onset-positive component (F (1, 8.06) = 0.041, p = .844) or the onset-negative component (F (1, 22.59) = 1.37, p = .255).
